# Global alteration of T-lymphocyte metabolism by PD-L1 checkpoint involves a block of de novo nucleoside phosphate synthesis

**DOI:** 10.1038/s41421-019-0130-x

**Published:** 2019-11-26

**Authors:** Nicolaos Jay Palaskas, Jacob David Garcia, Roksana Shirazi, Daniel Sanghoon Shin, Cristina Puig-Saus, Daniel Braas, Antoni Ribas, Thomas Glen Graeber

**Affiliations:** 1 Division of Hematology and Oncology, Department of Medicine, University of California Los Angeles, Los Angeles, CA 90095 USA; 2 Department of Molecular, Cellular, and Integrative Physiology, University of California Los Angeles, Los Angeles, CA 90095 USA; 3 Department of Molecular, Cellular, and Developmental Biology, University of California Los Angeles, Los Angeles, CA 90095 USA; 4 Department of Microbiology, Immunology, and Molecular Genetics, University of California Los Angeles, Los Angeles, CA 90095 USA; 5 Crump Institute for Molecular Imaging, University of California Los Angeles, Los Angeles, CA 90095 USA; 6 Metabolomics Center, University of California Los Angeles, Los Angeles, CA 90095 USA; 7 Department of Molecular and Medical Pharmacology, University of California Los Angeles, Los Angeles, CA 90095 USA; 8 Jonsson Comprehensive Cancer Center, University of California Los Angeles, Los Angeles, CA 90095 USA; 9 Division of Surgical Oncology, Department of Surgery, University of California Los Angeles, Los Angeles, CA 90095 USA

**Keywords:** Tumour immunology, Metabolomics, DNA metabolism, Cancer microenvironment, RNA metabolism

## Abstract

Metabolic obstacles of the tumor microenvironment remain a challenge to T-cell-mediated cancer immunotherapies. To better understand the interplay of immune checkpoint signaling and immune metabolism, this study developed and used an optimized metabolite extraction protocol for non-adherent primary human T-cells, to broadly profile in vitro metabolic changes effected by PD-1 signaling by mass spectrometry-based metabolomics and isotopomer analysis. Inhibitory signaling reduced aerobic glycolysis and glutaminolysis. A general scarcity across the panel of metabolites measured supported widespread metabolic regulation by PD-1. Glucose carbon fate analysis supported tricarboxylic acid cycle reliance on pyruvate carboxylation, catabolic-state fluxes into acetyl-CoA and succinyl-CoA, and a block in de novo nucleoside phosphate synthesis that was accompanied by reduced mTORC1 signaling. Nonetheless, exogenous administration of nucleosides was not sufficient to ameliorate proliferation of T-cells in the context of multiple metabolic insufficiencies due to PD-L1 treatment. Carbon fate analysis did not support the use of primarily glucose-derived carbons to fuel fatty acid beta oxidation, in contrast to reports on T-memory cells. These findings add to our understanding of metabolic dysregulation by PD-1 signaling and inform the effort to rationally develop metabolic interventions coupled with immune-checkpoint blockade for increased treatment efficacy.

## Introduction

Immune-checkpoint blockade targeting programmed cell death 1 (PDCD1/PD-1) and its ligand CD274 (PD-L1) has shown promise for the treatment of tumors of various histologies, with long-term responses and limited toxicities in a subset of patients^1^. T-cells found at the tumor margin can infiltrate and proliferate within the tumor upon successful immune-checkpoint blockade^2^. The tumor microenvironment presents obstacles to T-cell infiltration, and there is a growing appreciation for the metabolic restrictions imposed, such as competition with tumor cells for glucose^3^. Thus, it may be advantageous to couple immune-checkpoint blockade with metabolic interventions, and to include metabolic robustness in the engineering of cytotoxic T-cells^4^. Detailed knowledge of T-cell metabolism in different contexts would be a prerequisite for the rational development of such strategies.

The study of metabolism changed radically with the ability to do systems-level analyses using mass spectrometry^5^. Currently, there is no consensus regarding how to prepare non-adherent mammalian cell samples for metabolomics, and even less guidance specific for immune cells^6,7^. Metabolomic studies of human T-cells have used as many as 30 million cells per replicate^8,9^, an input requirement which may prohibit profiling rare T-cell subsets. A typical extraction protocol for intracellular metabolites includes: (i) separation of cells from media, (ii) a wash step to eliminate remaining contaminating media metabolites, (iii) quenching of metabolism and extraction of metabolites. Separation and washing can be done rapidly with adherent cells, but centrifugation requires prolonged exposure to the wash solution, which ideally should maintain physiological conditions while minimizing post-harvesting metabolic activity.

The purpose of this study was to broadly profile metabolic changes in human T-cells effected by PD-1 axis signaling using mass spectrometry-based metabolomics analyzing a panel of 155 polar and semi-polar metabolites. We simulated in vitro a tumor-directed attack by primed T-cells and used recombinant human PD-L1 protein to engage the immune-checkpoint, without the use of target cells and ensuing issues of cell separation and contaminating metabolites. To ensure profiling physiological-range conditions, antibody-based activation was adjusted using melanoma cell line antigen presentation as a guide. Our approach to minimize input requirements and optimize readout included screening several candidate wash solutions. As a reference control, we tested the ability of our assay parameters to identify expected metabolic changes induced by substitution of glucose by galactose in culture media. Finally, we incorporated [U-^13^C] glucose tracer experiments and analyzed carbon fate by steady-state isotopomer distributions of metabolites to infer relative pathway activities. This is particularly useful for non-linear pathways with multiple possible contributing pathways, such as the tricarboxylic acid (TCA) cycle^10^.

## Results

### Simulating an abortive T-cell tumor-directed attack

**Fig. 1:**
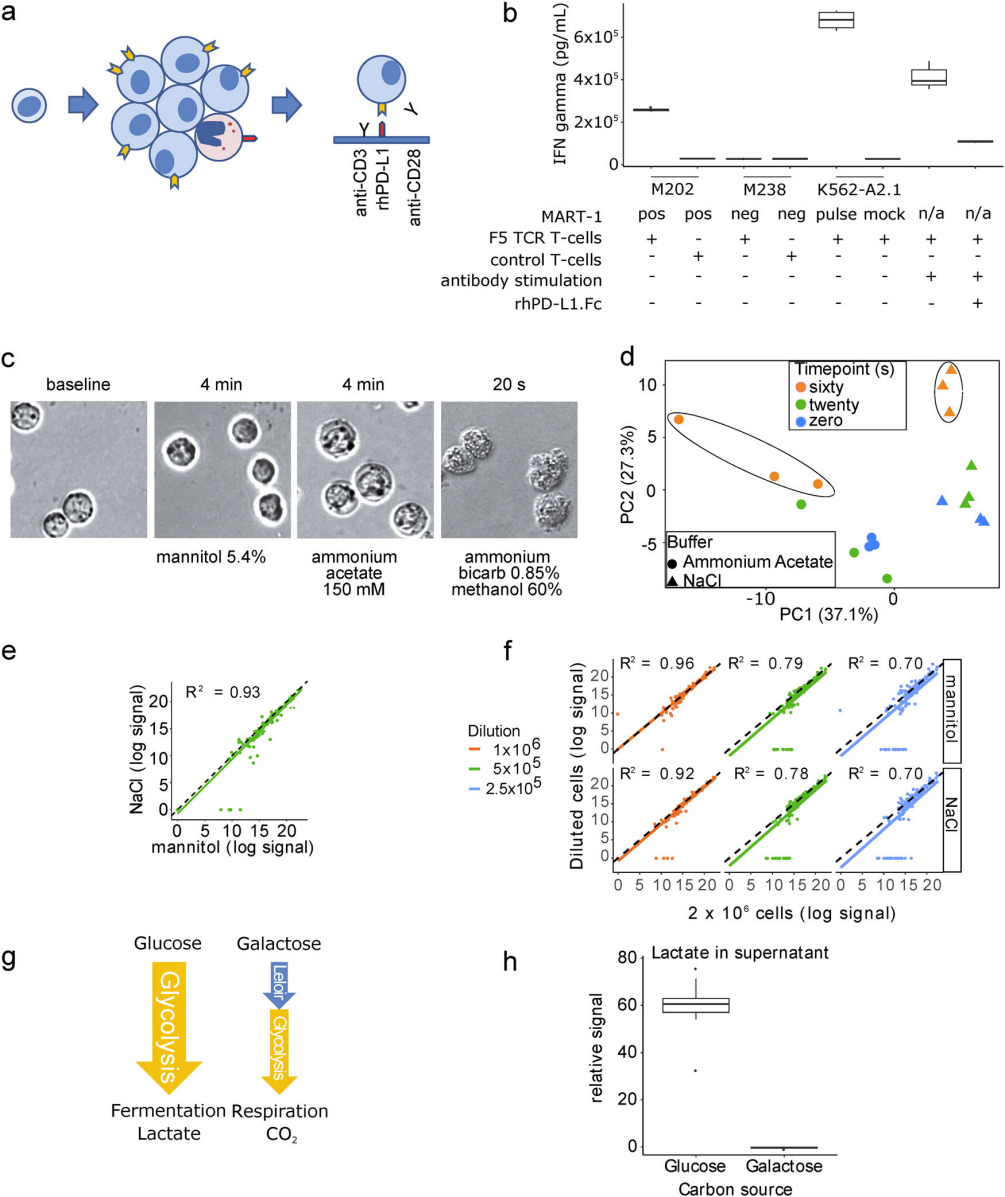
Development of a platform to interrogate PD-L1-induced changes by LC/MS metabolomics. **a** Schematic representation of T-cell treatment prior to metabolite extraction. PBMC are expanded using a clinical grade adoptive cell transfer protocol that leads to upregulation of PD-1 receptor on the surface of T-cells. Isolated T-cells are then seeded in plates with anti-CD3 and anti-CD28 antibodies with or without recombinant human PD-L1 (rhPD-L1). **b** IFN gamma ELISA of 24 h supernatants. T-cells bearing an exogenously expressed MART-1-specific T-cell receptor (F5 TCR) were co-cultured with M202 melanoma cells that present MART-1 via HLA-A2.1, M238 cells that do not, K562 cells with exogenous expression of HLA-A2.1 pulsed with MART-1_26–35_ peptide, or stimulated with anti-CD3 and anti-CD28 antibodies without target cells. Anti-CD3 and anti-CD28 antibody-based activation of T cells is comparable to levels of cell-based melanoma antigen presentation. **c** Light microscopy images show changes in cellular morphology with certain wash solutions. Jurkat cells were exposed for the indicated time intervals. The ammonium acetate iso-osmotic wash buffer provides little tonicity and causes considerable swelling of the cells. The ice-cold ammonium bicarbonate solution in 60% methanol causes cell changes on contact. In contrast, such changes are not noted with the mannitol solution. **d** Principal component analysis of 137 of 155 profiled metabolites demonstrates that the variance of intracellular metabolite measurements increases as a function of time when exposed to the ammonium acetate solution. Spontaneously immortalized 3T3 mouse embryonic fibroblasts were exposed to a 0.9% NaCl or 150 mM ammonium acetate solutions for the indicated times. The ellipses were added for emphasis. **e** Correlation of profiled metabolites between mannitol and NaCl wash solutions. Triplicates of 2 × 10^6^ Jurkat cells per condition. **f** Correlation of metabolite measurements of two-fold serially diluted samples. Values are corrected for the dilution factor. The dashed lines represent a perfect correlation. The solid colored lines are the best fit lines of the data. **g** Schematic model displaying the reduced flux of galactose metabolism ending with complete oxidation to CO_2_ compared to high glycolytic flux with glucose and production of lactate. **h** Supernatant lactate levels of activated primary human T-cells in glucose-containing or galactose-containing culture medium. Displayed are the summary results of three experiments, sampled from three replicate wells per condition (*n* = 9 vs. 9).

To simulate an abortive T-cell tumor-directed attack, we used stimulating antibodies, followed by recombinant human PD-L1 exposure in a plate-based system. Previously frozen PBMC were thawed and expanded for 7 days with induction of surface PD-1 (Supplementary Fig. S1) before T-cell isolation and treatment (Fig. 1a). Multi-parameter immuno-phenotyping of T-cell differentiation using this expansion protocol was previously published by our group^11^. We adjusted our treatment conditions to have the same cytokine-based output levels as a cell-based antigen presentation system. MART-1-specific transgenic human T-cells (F5 TCR) were co-cultured with peptide-presenting target cells and interferon gamma (IFNγ) release was used to gauge activation and inhibition. As expected, M202 melanoma and K562-A2.1 peptide-pulsed cells caused IFNγ release, contrary to mock-pulsed cells and M238 melanoma cells that do not present the peptide (Fig. 1b). At a similar level of activation using stimulating antibodies, the PD-L1 peptide efficiently inhibited IFNγ release (Fig. 1b).

### Optimization of metabolite collection and analysis

Next, we sought to find an appropriate wash solution for our intracellular metabolite extraction protocol. We reasoned that microscopy could provide a quick screen for drastic morphologic changes. Ammonium acetate, a common metabolite collection-protocol wash solution for adherent cells, caused Jurkat cells to swell over 4 min, while a methanol-based solution altered morphology on contact (Fig. 1c). To more formally assess the effect of ammonium acetate, we used adherent mouse embryonic fibroblasts (MEF) cells, because their exposure time to NaCl 0.9% (isotonic) and ammonium acetate solutions could be easily controlled. The variability of measurements with ammonium acetate increased as a function of exposure length (Fig. 1d). To overcome the osmotic swelling, we chose to proceed with an isotonic solution. Metabolite profiles of Jurkat cells using NaCl vs. mannitol isotonic wash solutions were highly correlated, with *R*^2^ = 0.93 (Fig. 1e), and with mannitol preserving the measurement of more metabolites. To co-optimize for a low cellular input amount that can quantitatively detect a substantial number of metabolites, we first assessed the ability of serial two-fold dilutions to predict the metabolite levels of the maximum cell number sample and found a good albeit decreasing correlation across the range of dilutions (Fig. 1f). Nonetheless, the number of reproducibly measured metabolite levels was highest at 5 × 10^5^ cells, although at the cost of detecting fewer metabolites. Further, our ability to confidently measure decreasing levels of metabolites of successive dilutions was best between 1 × 10^6^ and 5 × 10^5^ cellular inputs (Supplementary Fig. S2). Based on these comparisons, together with format considerations, we proceeded with 8.4 × 10^5^ cells per replicate, using a mannitol wash solution.

To test our parameters for metabolic extraction in the context of measuring a metabolic change, we activated T-cells in galactose-containing medium, which is known to decrease glycolytic flux and force cells to respire (Fig. 1g)^12^. As anticipated, lactate production was suppressed in galactose-containing medium (Fig. 1h). After 24 h, we detected higher intracellular levels of Leloir pathway intermediates UDP-hexose and hexose-phosphate with galactose treatment (Table 1). Aspartic acid was also elevated, consistent with recent reports of respiration driving aspartate production in proliferating cells^13,14^. Glycolytic intermediates were higher in the glucose condition, as was UDP N-acetylglucosamine, a reflection of glucose availability for the hexosamine pathway^15^. These findings fit expectations based on the literature.

**Table 1:** Intracellular metabolite level changes of activated human primary T-cells in culture medium containing glucose vs. galactose.

Compound	Fold change glucose vs. galactose	*p*-value	FDR	Pathway
UDP-hexose	−1.42	6.39 × 10^−5^	7.46 × 10^−04^	Leloir
Hexose-phosphate	−8.98	4.07 × 10^−4^	3.36 × 10^−03^	Both
Fructose-1,6-bisphosphate	9.22	8.37 × 10^−7^	2.34 × 10^−05^	Glycolysis
Glyceraldehyde-3-P	1437.38	9.12 × 10^−4^	4.91 × 10^−03^	Glycolysis
Dihydroxyacetone-P	583.69	6.54 × 10^−3^	2.41 × 10^−02^	Glycolysis
3-phosphoglycerate	2.75	4.90 × 10^−4^	3.61 × 10^−03^	Glycolysis
Phosphoenolpyruvate	1.24	7.23 × 10^−1^	8.04 × 10^−01^	Glycolysis
Pyruvate	−1.52	4.44 × 10^−1^	5.92 × 10^−01^	Glycolysis
Lactate	44.66	2.41 × 10^−6^	3.97 × 10^−05^	Glycolysis
UDP N-acetylglucosamine	16.26	1.46 × 10^−6^	2.92 × 10^−05^	Misc
Aspartic acid	−5.97	4.76 × 10^−4^	3.61 × 10^−03^	Misc

Summary of three experiments with each condition performed in triplicate

### Profiling of PD-L1 checkpoint-induced metabolic changes reveals a block in de novo nucleoside phosphate synthesis

**Fig. 2:**
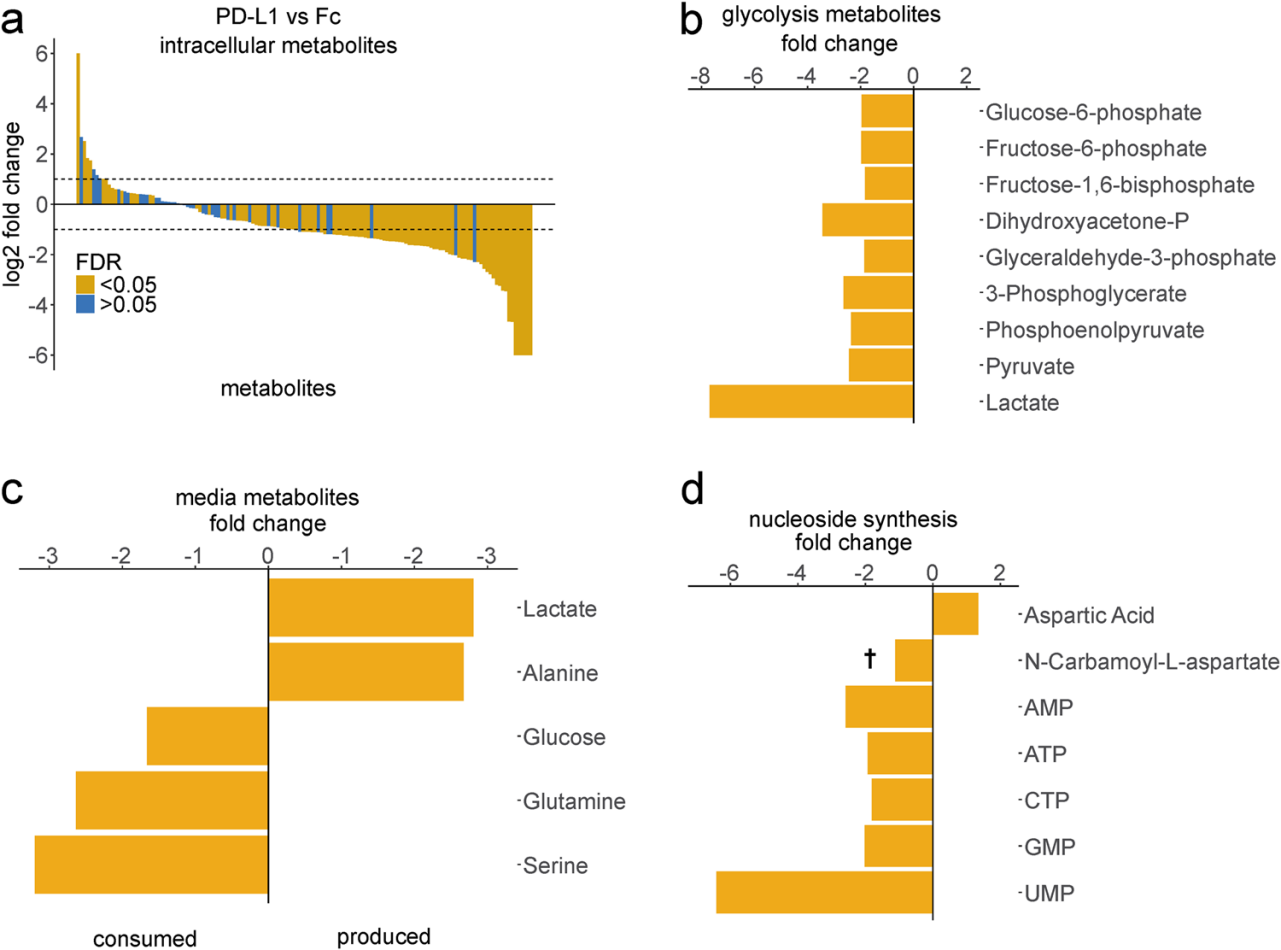
Relative levels of intracellular and supernatant metabolites in activated vs. PD-L1-inhibited T-cells. **a** Overview of all profiled intracellular metabolites as measured by mass spectrometry. Positive values indicate higher levels in the treated condition (PD-L1). Dotted lines represent a two-fold change on the log_2_ scale. Values are capped at 100-fold. **b** Intracellular glycolysis pathway intermediates are lower in inhibited cells. **c** Compared to Fc control cells, less lactate and alanine are produced, and less glucose, glutamine, and serine are consumed. **d** Nucleoside phosphates are reduced and aspartic acid levels are increased by PD-L1. FDR: false discovery rate. †: FDR > 0.05. All values represent four experiments with triplicates of each condition and have FDR < 0.05 unless indicated otherwise.

Next, we interrogated metabolic changes caused in primary human T-cells by PD1 axis signaling. After 72 h of treatment, we adequately measured 146 of 155 intracellular metabolites of our panel, and found most to be reduced by treatment (Fig. 2a). Intracellular levels of glycolytic intermediates were reduced (Fig. 2b), as were lactate levels in media (Fig. 2c). Consumption of glutamine and serine were also reduced (Fig. 2c). Relative levels of nucleoside phosphates were decreased (Fig. 2d).

**Fig. 3:**
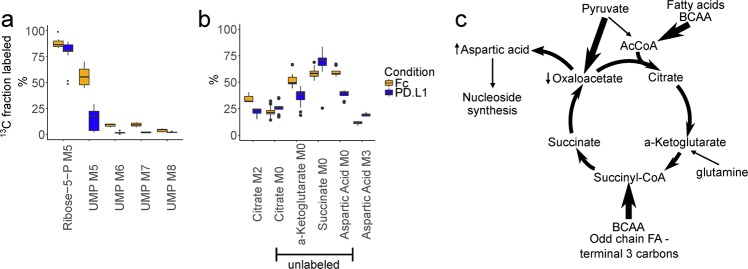
[U-^13^C] glucose tracing shows differences in metabolite contribution to the TCA cycle and decreased de novo nucleoside phosphate synthesis upon PD-L1 inhibition. **a** Selected isotopomers of uridine monophosphate and ribose-5-phosphate that represent the contribution of fully labeled ribose-5-phosphate (UMP M5) plus partially labeled aspartic acid (UMP M6–7). **b** Selected isotopomers of TCA metabolites used to infer metabolite contributions to the TCA cycle, expressed as the percentage of all isotopomers detected for the respective compounds. M0: unlabeled. M2: Two heavy carbons from glucose. M3: Three heavy carbons from glucose. **c** Schematic of inferred relative contributions to the TCA cycle in PD-L1-treated cells. Thick arrows represent more relative contribution of metabolites in the PD-L1-treated cells compared to the Fc control.

To probe further, we performed isotopomer analysis. This showed that despite similar incorporation of heavy carbons from [U-^13^C] glucose into ribose, very little label was incorporated into nucleoside phosphates with PD-L1 treatment (Fig. 3a and Supplementary Fig. S3). Isotopomer analysis further showed differential carbon labeling of Krebs cycle intermediates (Fig. 3b and Supplementary Fig. S4). Based on these carbon tracing changes, we inferred a model of fluxes into the cycle. PD-1 signaling results in increased reductive carboxylation of pyruvate, anaplerosis of the cycle at acetyl-CoA and succinyl-CoA that could come from fatty acids and branched chain amino acids, less anaplerosis from glutamine, and less nucleoside synthesis despite higher relative levels of aspartate (Fig. 3c).

**Fig. 4:**
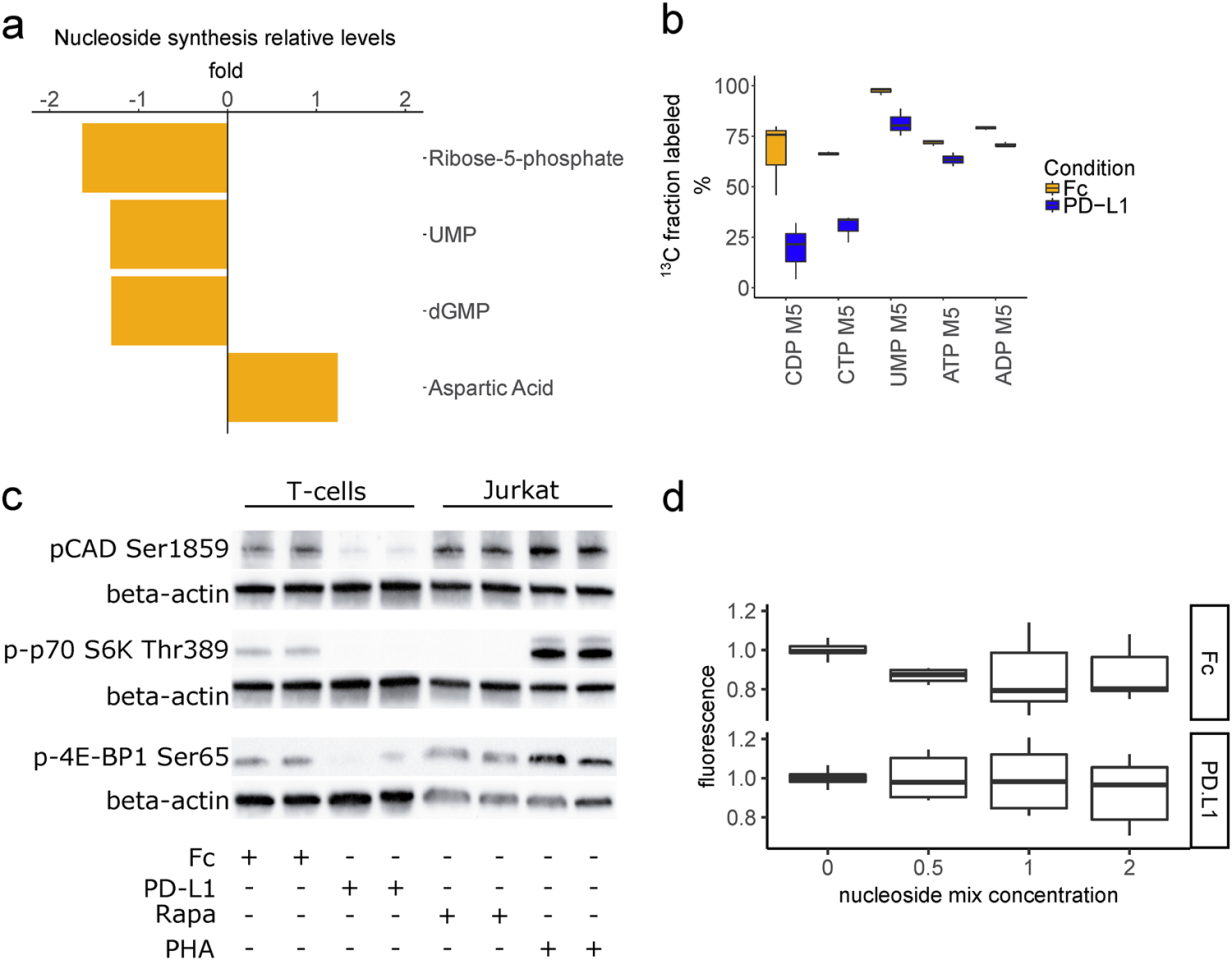
PD-L1 causes a block in nucleoside phosphate synthesis at 24 h. **a** Relative levels of nucleoside phosphates and aspartic acid at 24 h of treatment. **b** Statistically significantly changing ^13^C incorporation into nucleoside phosphates from [U-^13^C] glucose via ribose-5-P, as reflected in the their M5 isotopomers. All measurements have a FDR < 0.05 but the fold-reduction is larger for pyrimidines than for purines. **c** Western blot of phosphorylation status of mTORC1 targets. T-cells were treated for 48 h as indicated. Jurkat cells were treated for 2 h. PHA: phytohemagglutinin 5 µg/mL. Rapa: rapamycin 20 nM. **d** Metabolic activity (alamar blue resazurin reduction) assay of Fc and PD-L1-treated T-cells with or without nucleoside cocktail supplementation for 48 h. Summary of three experiments performed on separated days in triplicates (*n* = 9 per condition).

We questioned whether the block in nucleoside phosphate synthesis was an early event. At a 24 h time-point, the landscape of metabolic changes was consistent but more modest than at 72 h (Supplementary Fig. S5). Among relative level changes caused by PD-L1 treatment with an FDR < 0.05, we found reduced ribose-5-P and UMP, and increased aspartic acid (Fig. 4a). The inhibited cells incorporated less ^13^C from ribose-5-P into nucleoside phosphates, as reflected in their M5 isotopomers. At this early timepoint, the fold reduction was larger for pyrimidines than purines (Fig. 4b). The mechanistic target of rapamycin complex 1 (mTORC1) has been shown to regulate nucleoside phosphate synthesis^16,17^. We probed phosphorylation sites on downstream targets of mTORC1, including carbamoyl-phosphate synthetase 2, aspartate transcarbamylase, and dihydroorotase (CAD), and found decreased mTORC1 activity with PD-L1 treatment (Fig. 4c). In certain cellular contexts, isolated deficiency of nucleoside phosphate synthesis can be rescued by providing exogenous downstream substrates, including nucleosides^16–18^. However, in this checkpoint context, we could not document a rescue of proliferation in PD-L1-treated cells supplemented with exogenous nucleosides (Fig. 4d and Supplementary Fig. S6).

### Inhibition of mTOR partially phenocopies the PD-L1-mediated block in de novo nucleoside phosphate synthesis

**Fig. 5:**
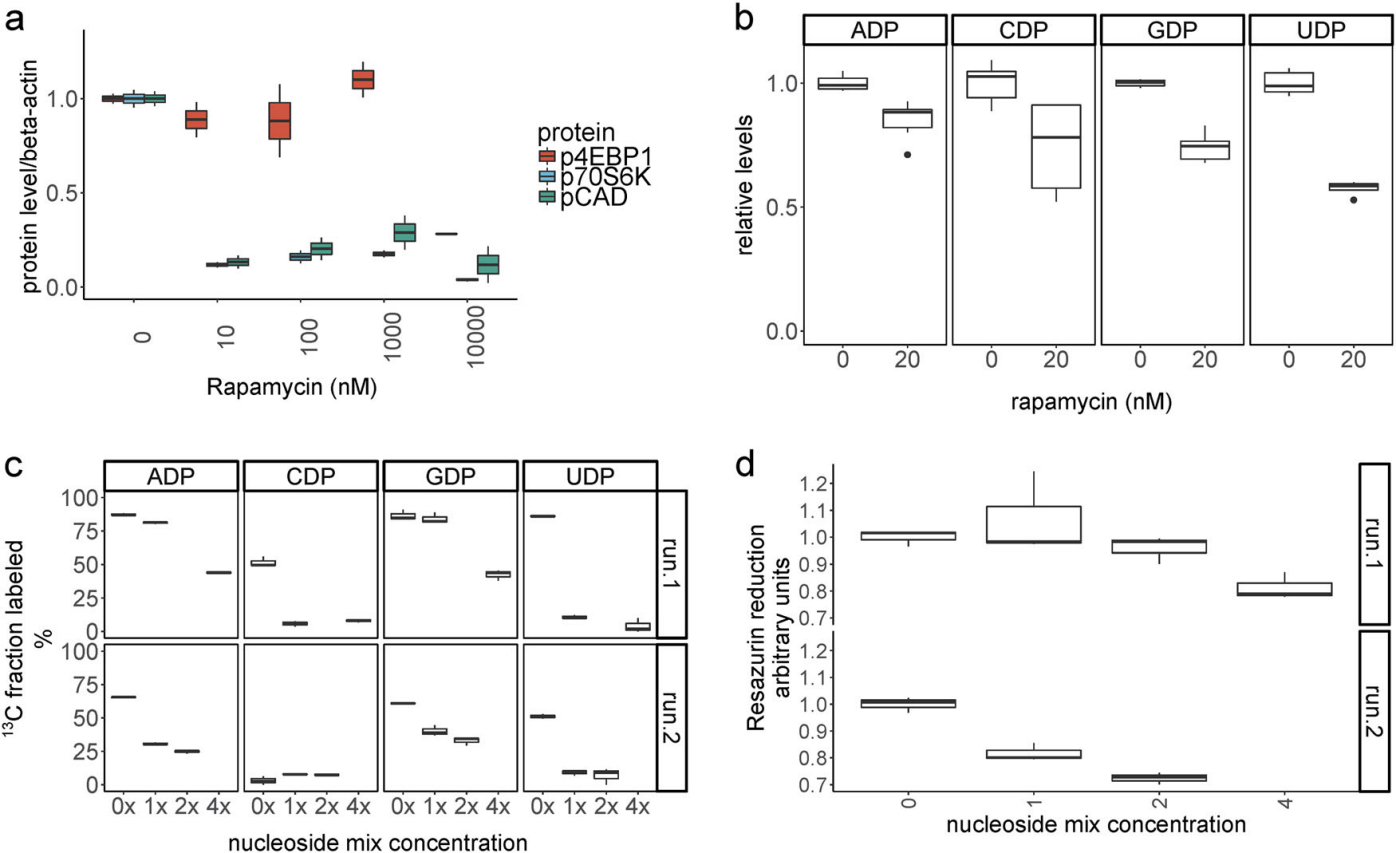
Nucleoside supplementation does not circumvent mTOR inhibition. **a** T-cells were treated with the indicated concentrations of rapamycin for 48 h and levels of mTORC1 phosphorylation targets were assayed by western blot. **b** Rapamycin treatment reduces relative intracellular levels of nucleoside phosphates. Summary of two experiments run in triplicates (*n* = 6, 6) assayed at a 48 h time-point. **c** Supplementation of unlabeled nucleosides results in a decrease of glucose-derived heavy carbon label incorporation into nucleoside phosphates at 48 h. Results of two experiments with all conditions run in triplicate. **d** Metabolic activity (alamar blue resazurin reduction) assay of T-cells treated with 20 nM rapamycin for 48 h and additionally treated with the indicated concentrations of nucleoside cocktail.

We questioned whether the block in pyrimidine nucleoside phosphate synthesis could be solely attributed to mTORC1 inhibition, or whether additional PD-L1-activated mechanisms were responsible for the magnitude of the phenotype and its refractoriness to reversal by exogenous nucleoside administration. To address this question we similarly stimulated and expanded T-cells for 7 days and then treated for 48 h with rapamycin, a known inhibitor of mTORC1 and an immunosuppressant used clinically^19^. We found that low doses of rapamycin were sufficient to block phosphorylation of p-70 S6 kinase and its target phosphorylation site on CAD, although phosphorylation of 4E-BP1 was only reduced at much higher doses (Fig. 5a and Supplementary Fig. S7). We next measured intracellular nucleoside phosphate levels and found lower levels upon rapamycin treatment (Fig. 5b), yet the effect was smaller than with PD-L1 treatment for 72 h (Fig. 2d). We confirmed that exogenous unlabeled nucleosides could be incorporated in the intracellular metabolite pool by measuring the fraction of [U-^13^C] glucose-derived label. Glucose-derived heavy carbon labeling could be competitively depleted by increasing concentrations of unlabeled nucleosides (Fig. 5c). However, similarly to the PD-L1 treatment, mTOR inhibition could not be circumvented by the administration of the nucleoside cocktail, as measured by the metabolic activity (resazurin reduction capacity) of the treated T-cells (Fig. 5d).

## Discussion

Our motivation to study human T-cell metabolism led us to develop an in vitro platform where cells can be activated and treated with ligands, drugs, or different nutrient conditions, and metabolic changes can be measured by LC/MS, with the use of an extraction protocol optimized for T-cells. This approach enabled us to exhibit a landscape of metabolic changes caused by PD-1 checkpoint engagement. Our study is unique, in that we have used a cell-based antigen presentation system as a guide for relevant in vitro artificial stimulation, and used [U-^13^C] glucose, to infer pathway activities and substrate utilization in PD-L1-treated primary human T-cells. This strategy allowed us to uncover a PD-L1-induced block in de novo nucleoside phosphate synthesis, although it remains an in vitro study of a single signaling pathway and cannot fully capture the complexities of the tumor microenvironment.

There are several proposed methods for metabolite extraction of non-adherent cells. We found isotonic solutions to be the most appropriate for washing (Fig. 1c, d) and chose to work with mannitol because it is compatible with both LC/MS and capillary electrophoresis technologies^20^. Early in our investigation we evaluated a filter-based method originally applied to microbial extraction that did not require pumps and automation^21^, but despite experimenting with several filter types and sizes we found filter clogging to be an issue. This limitation and reports of suboptimal recovery of metabolites from filters^6^ led us to prioritize a centrifugation-based strategy. A methanol-based wash solution^22^ distorted cells on contact (Fig. 1c) and has been reported to cause leakage of metabolites^6^, leading us to consider this option no further. Ammonium acetate is a common metabolite extraction wash solution because it is volatile and should not contribute to ion suppression after evaporation during sample preparation. A study with primary focus on lipidomics in Jurkat cells concluded that ammonium acetate is appropriate for human non-adherent cell metabolomics^7^. In our work, we found that it causes cells to swell. Penetration of mammalian cells by ammonium salts of weak acids has been studied^23^. More importantly, we found metabolite variability increased as a function of time exposed to ammonium acetate (Fig. 1d). However, we analyzed polar metabolites and do not know whether the lipid compartment is similarly affected.

We found that PD-1 inhibitory signaling shifts metabolism away from aerobic glycolysis and glutaminolysis (Fig. 2b, c), and forces the cell to utilize alternative substrates to feed the TCA cycle (Fig. 3b, c), in agreement with previous reports^9,24^. The single other mass spectrometry-based metabolomics study related to PD-1 signaling^9^ reported a block in the uptake and utilization of branched chain amino acids (BCAA), based on relative levels of intracellular and extracellular valine, and intracellular levels of the downstream metabolite 4-methyl-2-oxopentanoate. Induction of carnitine palmitoyltransferase I expression and increased mitochondrial spare respiratory capacity were also demonstrated, reminiscent of findings in murine T-memory cells^25^. In contrast to T-memory cells, lysosomal acid lipase (LAL) was not increased while adipose triglyceride lyase (ATGL) protein levels were. ATGL appears dispensable for T-cell memory^25^. We showed that unlabeled carbons enter the TCA cycle as acetyl-CoA (Fig. 3c), which does not support the idea of PD-L1-treated cells engaging in “cell-intrinsic lipolysis”. This term refers to the futile diversion of glucose-derived carbon into triglyceride synthesis by T-memory cells, only to be mobilized from lysosomes to re-enter the TCA cycle^25^. Thus, fatty acid beta-oxidation appears qualitatively different between PD-1-signaling cells and T-memory cells. Future studies are required to elucidate whether these early metabolic differences between PD-1-signaling cells and T-memory cells are causally linked to their distinct epigenetic profiles and potential for reinvigoration^26,27^. Considering the block of BCAA catabolism reported with PD-1 signaling^9^, the increase of unlabeled carbons we documented entering the TCA cycle at succinyl-CoA (Fig. 3c) must be attributed to the terminal three carbons of odd-chain fatty acids alone. However, our study does not provide the fractional contribution of each substrate to definitively confirm a block in BCAA catabolism, nor can we discount effects that our different treatment conditions could have had on those fractions.

For the first time, we demonstrated that PD-1 signaling results in a block of nucleoside phosphate de novo synthesis. This was more notable for pyrimidines at an early, 24 h timepoint (Fig. 4b). The difference in timing is consistent with pyrimidine de novo synthesis being controlled rapidly by mTORC1 phosphorylation of CAD and the control of purine synthesis being regulated through slower transcriptional mechanisms^16,17^. Indeed, we observed less phosphorylation of canonical mTORC1 targets and CAD (Fig. 4c). Activated T-lymphocytes are dependent on de novo purine and pyrimidine synthesis for proliferation and survival, and nucleotides regulate their cell cycle^28^. Nonetheless, a cocktail of purines and pyrimidines by itself was not sufficient to rescue the proliferation of PD-L1-treated cells (Fig. 4d). Additionally, mTOR inhibition by rapamycin treatment only partially phenocopied the block in nucleoside phosphate de novo synthesis and incorporation of glucose-derived heavy carbons, even though the phosphorylation of CAD and p-70 S6K was effectively inhibited (Fig. 5a). On the one hand, this suggests that S6K activity is not the only determinant of de novo pyrimidine and purine synthesis, although required for full capacity. Rapamycin-resistant T-cell proliferation has been documented in the context of strong T-cell receptor stimulation^19^, which would require such residual ability to synthesize RNA and DNA building blocks. On the other hand, even the partial inhibition by rapamycin was not circumventable by the provision of exogenous nucleosides. In fact, this intervention appeared to be further deleterious to the metabolic fitness of the cells (Fig. 5d). Possibly, this is an indication of substrate level negative feedback^29^. While we did not identify an effective maneuver to clearly define the role of mTOR in the context of PD-L1 signaling, conceivably there may be a combination of individual nucleoside concentrations that would have a positive effect. Based on the more profound inhibition with PD-L1 compared to rapamycin it is tempting to invoke other nodes of metabolic regulation, in addition to the central role and multiple functions described for mTOR in T-cell biology^30^. PD-1 signaling also inhibits AKT, another central regulator of proliferation and metabolism^24^. Furthermore, PD-1 inhibits the cell cycle through upregulation of p27^kip1^, in contrast to the PI3K-dependent downregulation of p27^kip1^ described in rapamycin-resistant T-cell proliferation^19,31^.

Future in vivo studies with transgenic animals will likely be required to expand on our findings and define which metabolic interventions can be coupled with immunotherapies for increased therapeutic efficacy.

## Materials and methods

### Cell lines and culture

Peripheral blood mononuclear cells (PBMCs) were obtained from a healthy donor by leukapheresis under UCLA IRB#10-001598. M202 and M238 were established from patient biopsies with informed consent from all subjects under UCLA institutional review board approval IRB#02-08-067^32^. The K562-A2.1 cell line was a kind gift of Dr. Cedric Britten^33^. Human PBMCs, Jurkat cells, M202 and M238 melanoma cells were cultured in RPMI 1640 with glutamine and supplemented with 10% fetal calf serum (FCS) and 1% streptomycin, penicillin, and fungisome antibiotic cocktail (100x SPF, 10,000 units/mL penicillin, 10,000 µg/mL of streptomycin, and 25 µg/mL Amphotericin B). Immortalized MEF were cultured in DMEM with 10% FCS and 1% SPF. MEFs were immortalized as previously described^34^.

### T-cell-directed expansion of PBMCs

Human PBMCs were expanded toward the T-cell lineage as previously described^11^ using 10% FCS instead of human AB serum. Medium acidity was monitored and fresh medium added every 2 days to maintain a concentration of 7 × 10^5^ cells/mL.

### T-cell activation and treatment with PD-L1

Twelve-well non-tissue culture-treated plates (Corning #351143) were coated at 4 °C overnight with 1 µg/mL anti-CD3 antibody (BD biosciences 555329) and 1.5 µM human IgG Fc fragment (Jackson Immunoresearch #009-000-008) or 1.5 µM recombinant human Fc-tagged PD-L1 (rhPD-L1.Fc) (Sino Biological #10084-H02H) in 1.2 mL PBS per well. The plate was washed twice with 2 mL PBS and blocked for 1 h at room temperature with 1.5 mL human serum albumin 2.5% in phosphate-buffered solution (PBS), followed by two more washes with PBS. T-cells previously expanded for 7 days were isolated by immunomagnetic negative enrichment kit (ThermoFisher #11344D) and seeded in triplicate wells at 8.4 × 10^5^cells in 1.2 mL glucose-free RPMI (ThermoFisher #11879020) supplemented with 10 mM [U-^13^C] glucose (Cambridge Isotope Laboratories #CLM-1396-PK), 10% dialyzed fetal calf serum, 1% SPF, and 1 µg/mL anti-CD28 antibody (BD biosciences #555725). For 72-h treatments, the treatment medium was refreshed 24 h prior to harvest.

### T-cell metabolite extraction protocol

Treated T-cells were harvested into microcentrifuge tubes and centrifuged at 500×G-force for 4 min. The medium was collected and stored at −80 °C until further processing. Ice-cold 5.4% mannitol wash solution was added, 1 mL to each pellet, and centrifuged with the same parameters at 4 °C. Subsequent steps were performed on ice with solutions ice-cold. The wash solution was removed and sequentially 250 µL methanol, 250 µL water, and finally 250 µL chloroform were added, vortexing briefly between steps. The mixture was centrifuged at 16,000×G-force for 5 min. The top polar phase was collected into glass chromatography vials and stored at −80 °C until further processing. Interphase protein was measured by BCA assay kit (ThermoFisher #23225). The polar phase, 20 µL of the supernatants, and three mock-extracted controls per run (polar phase of extraction mixture without cells) were evaporated with an EZ2-Elite centrifugal evaporator for 80 min on HPLC setting with 30 °C maximum temperature. The samples were block-randomized and stored at −80 °C prior to recovery and submission of 1/10 of each replicate for mass spectrometry. Additional extraction methods tested are described in the supplemental methods.

### Co-culture of transgenic T-cells with melanoma and K562 targets

Transgenic MART-1-specific TCR-bearing T-cells (F5 cells) were co-cultured at 1:1 effector to target ratio for 24 h with melanoma, or either pulsed (MART-1_26–35_) or non-pulsed K562-A2.1 cells in 24-well plates with 1 × 10^6^ cells in 1 mL as previously described^35^. IFNγ release was assayed by ELISA (ThermoFisher #88-7316-77).

### Western blotting

Primary antibodies used were Phospho-CAD (Ser1859) Antibody #12662, Phospho-p70 S6 Kinase (Thr389) (108D2) Rabbit mAb #9234, Phospho-4E-BP1 (Ser65) (174A9) Rabbit mAb #9456, and β-Actin (D6A8) Rabbit mAb #8457 from Cell Signaling.

### Data analysis

Mass spectrometry metabolomics data were mean-normalized between runs. Significant changes were calculated using a two-sided Student’s *t*-test assuming unequal variance. *P*-values were adjusted for multiple hypothesis testing using Benjamini and Hochberg’s method of false discovery rate (FDR). Analysis was performed in R^36^.

## Supplementary information

Supplemental Materials

## Supplementary information

**Supplementary Information** accompanies the paper at (10.1038/s41421-019-0130-x).
