# Supplementary material for: Global alteration of T-lymphocyte metabolism by PD-L1 checkpoint involves a block of de novo nucleoside phosphate synthesis
Source: Cell Discov. 2019 Nov 26;5:62. doi: 10.1038/s41421-019-0130-x (PMC6877514; doi:10.1038/s41421-019-0130-x)
Supplement: Supplementary file 1 — Supplemental Materials [file 41421_2019_130_MOESM1_ESM.docx]

## Supplemental methods

### Timed exposure to wash solutions - Jurkat cell morphology. Related to Figure 1c.

Jurkat T-cell leukemia cells were seeded into poly-D-Lysine coated 96-well plates at 2 x 10^5^ cells per well in cell culture medium and centrifuged at 30 x G-force for 1 minute with the brake off. The plate was placed on a Nikon eclipse Ti microscope stage. One well at a time, the cell culture medium was aspirated and replaced with the indicated ice-cold wash solutions. Images were acquired at the indicated times.

### Wash solution time-course with adherent cells for metabolomics. Related to Figure 1d.

Spontaneously immortalized mouse embryonic fibroblasts were seeded in triplicate at 2.5 x 10^5^ cells per 9 cm^2^ and incubated overnight to allow for attachment. Procedures were performed with the plate on ice. The culture medium was aspirated one replicate at a time and washed with either ice-cold 150 mM ammonium acetate aqueous solution or 0.9% (w/v) NaCl solution. The wash solution was aspirated either immediately, at 20 seconds, or at 60 seconds. Metabolites were extracted directly from the plate with 1 mL ice-cold 80% (v/v) methanol/H_2_O. The cells were scraped and the content of the plate transferred to a 1.5 mL plastic disposable centrifuge tube and placed first on dry ice and then into a -80° freezer until further processing.

### Serial dilution of Jurkat cells and centrifugation-based extraction. Related to Figure 1f.

Jurkat cells were serially two-fold diluted to give 2 x 10^6^, 1 x 10^6^, 0.5 x 10^6^, and 0.25 x 10^6^ cells per replicate. The cells were separated from culture medium by centrifugation at 500 x G-force for 4 min followed by aspiration. Mannitol or NaCl ice cold solutions of 1 mL volume were added and followed by a second centrifugation using the same parameters at 4° C. After aspirating the wash solution the cells were immediately extracted using 250 µL ice-cold methanol, followed by 250 µL diH_2_O, and then 250 µL chloroform, vortexing briefly in between steps. The mixture was centrifuged at top speed for 5 min at 4° C and the upper aqueous phase was collected into a glass chromatography vial and stored at -80° C until further processing as described for T-cell metabolomics in the main methods.

### Carbon source experiments. Related to Figure 1g-h and Table 1.

Glucose-free RPMI 1640 (ThermoFisher #11879020) was supplemented with 10 mM glucose or galactose, 10% dialyzed fetal calf serum and 1% SPF. Cells were seeded in activating conditions in 12-well plates at 8.4 x 10^5^ cells per 1.2 mL culture medium in triplicate for each carbon source. After 24 hours, media was collected and intracellular metabolites extracted as per the T-cell metabolite extraction in the main methods.

### Liquid chromatography and mass spectrometry

Metabolites were recovered in 50 μL 70% ACN and 5 μL of this solution used for mass spectrometer-based analysis performed on a Q Exactive (Thermo Scientific) coupled to an UltiMate 3000RSLC (Thermo Scientific) UHPLC system. Mobile phase A was 5 mM NH4AcO, pH 9.9, B was ACN, and the separation achieved on a Luna 3u NH2 100A (150 × 2.0 mm) (Phenomenex) column. The flow was kept at 200 μL/min, and the gradient was from 15% A to 95% A in 18 min, followed by an isocratic step for 9 min and re-equilibration for 7 min. Metabolites we detected and relatively quantified as area under the curve (AUC) based on retention time and accurate mass (≤ 3 p.p.m.) using TraceFinder 3.3 (Thermo Scientific) software.

**Cell quantitation assay. Related to Figure 4d and Supplementary Figure S6.**

Plates were coated as per the main methods using 100 µL/well of coating solution in 96-well format. Day 7 isolated T-cells were seeded at 7x10^4^ cells/well in RPMI 1640 with 2 mM glutamine, 10%FBS, 1% SPF, and the indicated concentrations of nucleoside mixture (EmbryoMax® Nucleosides (100X), MilliporeSigma #ES-008-D). Surrogate measurements of cell number were performed after 48 hours by fluorescence of either resazurin reduction to resorufin (alamarBlue®, BIO-RAD #BUF012A), or DNA (CyQUANT™ Direct Cell Proliferation Assay, ThermoFisher # C35011) .

**Immunophenotyping. Related to Supplementary Figure S1.**

At least 3 x 10^5^ cells per condition were stained with anti-PD-1 antibody (Biolegend #329935), or isotype control (Biolegend #400132), after blocking with fetal bovine serum. Data were acquired at the Jonsson Comprehensive Cancer Center flow cytometry core on the LSR II Flow Cytometer and analyzed by Flowjo (Tree Star Inc.).

## Supplemental figures

**
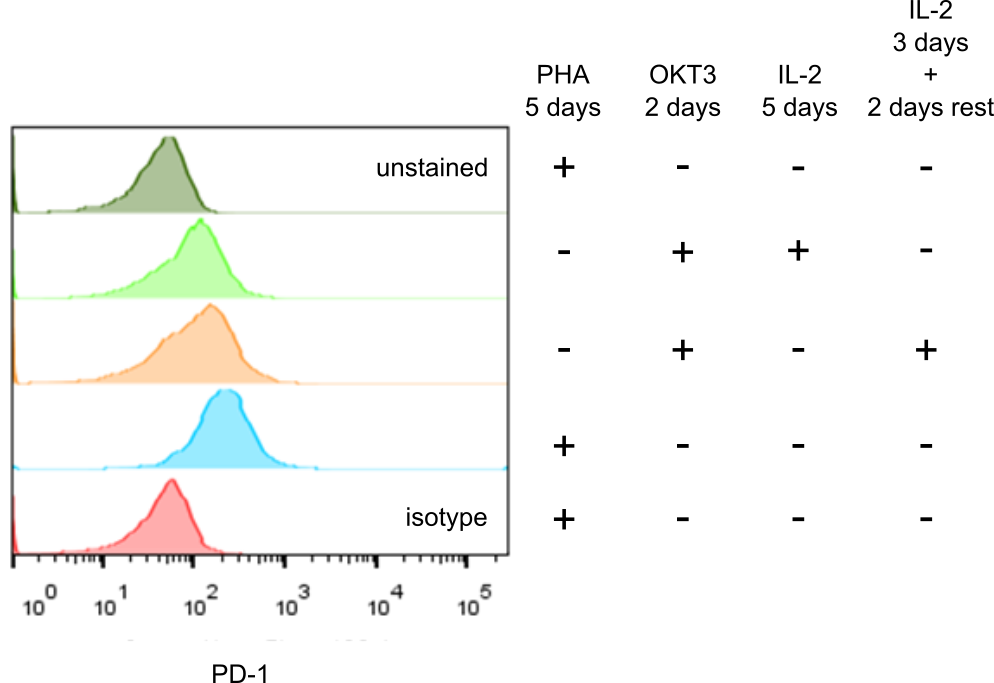
**

### Supplementary Figure S1. Related to Fig. 1a. Surface PD-1 induction by expansion conditions.

PBMC were thawed and expanded in the indicated conditions and stained for PD-1 surface expression, measured by flow cytometry. PHA: phytohemaglutinin 5 µg/mL. OKT3: soluble anti-CD3 antibody 50 ng/mL. IL-2: 300 IU/mL. Tumeh et al. provide multicolor immunophenotyping data using this expansion protocol^11^.


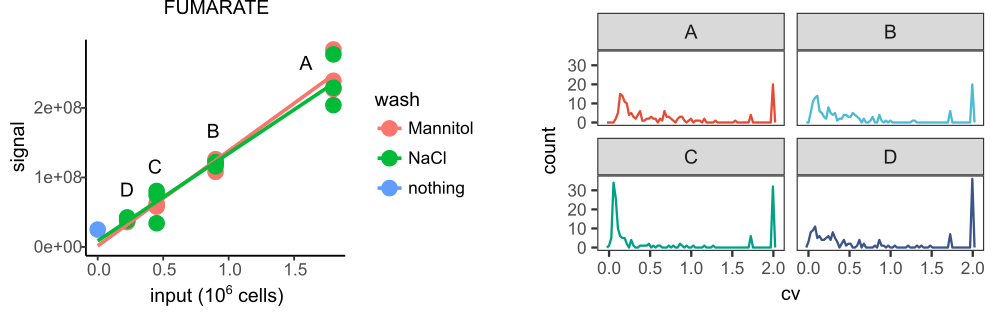


| Input (number of cells) | A  2x10^6^ | B  1x10^6^ | C  5x10^5^ | D  2.5x10^5^ |
| --- | --- | --- | --- | --- |
| # compounds with coefficient of variation < 0.3 | *67* | **74** | **98** | *70* |
| # compounds detected | *135* | **135** | **123** | *119* |
| # compounds decreasing with p<0.05 vs A |  | *52* |  |  |
| # compounds decreasing with p<0.05 vs B |  |  | **70** |  |
| # compounds decreasing with p<0.05 vs C |  |  |  | *63* |

### Supplementary Figure S2. Related to Fig. 1f

Left: The metabolite fumarate was measured well across the range of two-fold dilutions using either mannitol or NaCl wash solutions prior to extraction. A: 2x10^6^ cells, B: 1x10^6^ cells, C: 5x10^5^ cells, D: 2.5x10^5^ cells. Right: Frequency of metabolites plotted against their coefficient of variation (CV). Undetected metabolites were arbitrarily set to a CV of 2, for visualization. Condition C has the most precise measurements, but with fewer total metabolites detected compared to the higher cell inputs. The metabolite extraction was performed with a mannitol wash solution step. Bottom: Tabular summary of statistics. The highest number of confidently decreasing metabolites (n = 70) was measured between conditions B and C. Student’s one-sided T-test.

**
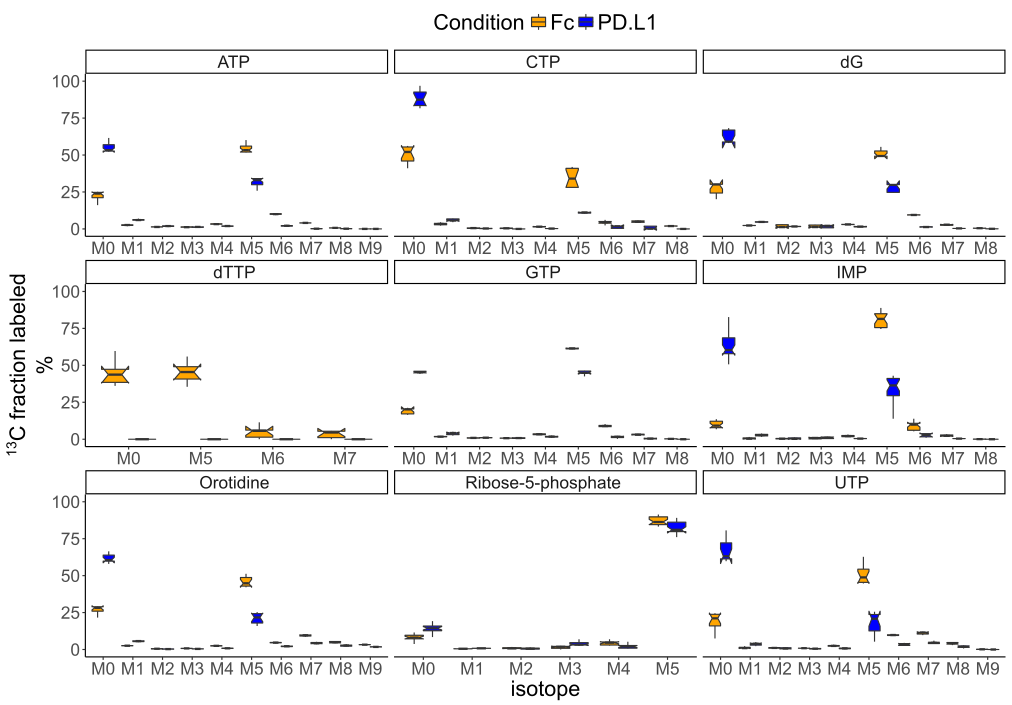
**

### Supplementary Figure S3. Related to Fig. 3a.

De novo nucleoside phosphate synthesis from [U-^13^C] glucose is reduced in PD-L1 treated T-cells, as seen by less heavy carbon incorporation.

**
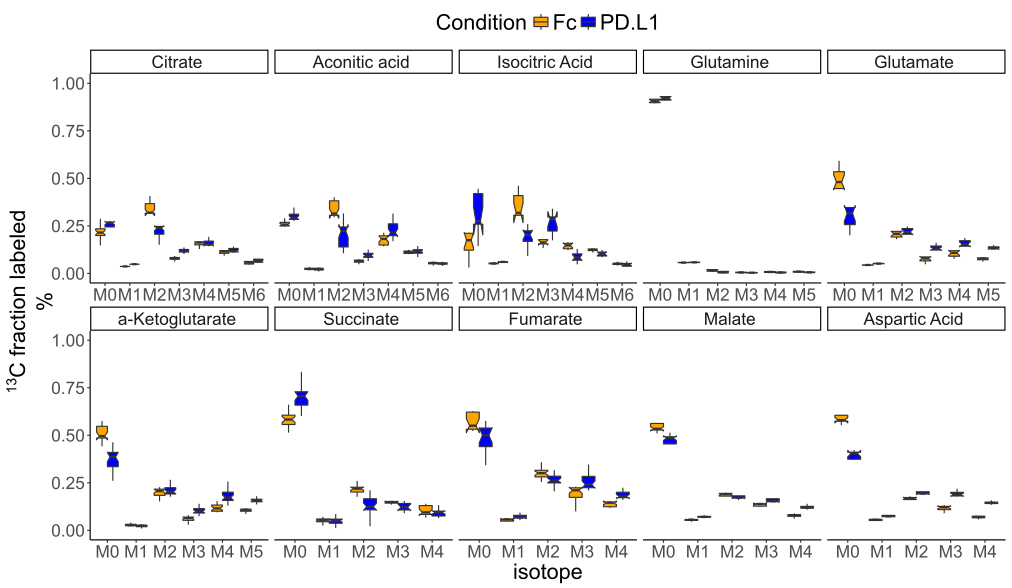
**

### Supplementary Figure S4. Related to figure 3b-c.

Isotopomers of [U-^13^C] glucose-labeled tricarboxylic acid cycle related metabolites. PD-L1 treatment results in fewer labeled 2-carbon units in citrate M2 from acetyl-CoA of glucose origin. A flipped ratio of unlabeled a-ketoglutarate M0 is consistent with less influx of unlabeled glutamine. Another reversal of succinate M0 is consistent with increased influx of unlabeled carbons at succinyl-CoA. An increase of M3 label in aspartic acid, malate, and fumarate compared to succinate is consistent with pyruvate carboxylation.

**
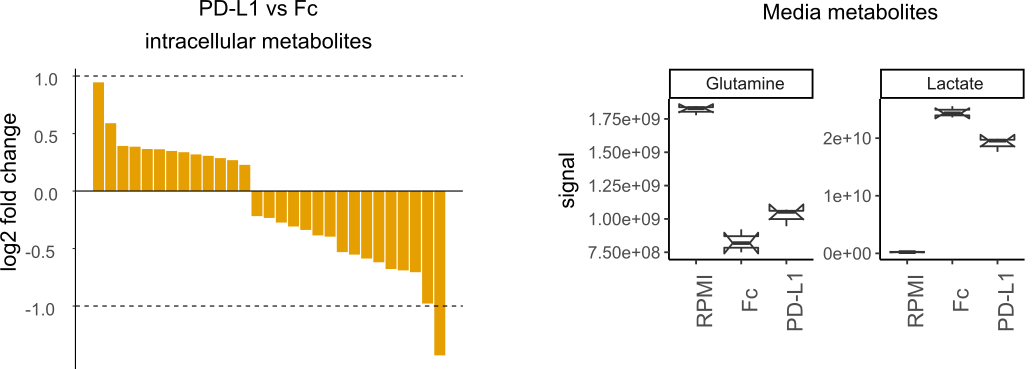
**

### Supplementary Figure S5. Related to Figure 4. Metabolic changes at 24 hours.

Left: All intracellular metabolites with a FDR ≤ 0.05 (n = 29). Fold changes are modest compared to Fig. 2a. Right: Butterfly plot of decreased glutamine consumption and lactate production with PD-L1 treatment, measured in cell culture media by LC/MS. The notches/wings are calculated as 1.58 * interquartile range / sqrt(n) and approximate a 95% confidence interval. RPMI: unspent culture medium.

**
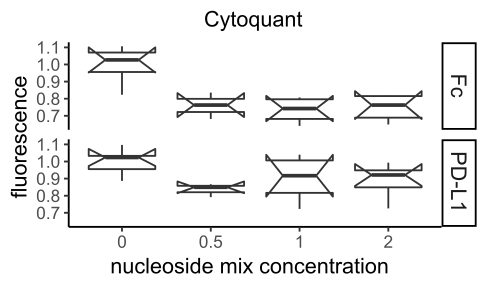
**

### Supplementary Figure S6. Related to Figure 4d.

Cell quantitation using DNA fluorescence in T-cells treated with PD-L1 and the indicated concentrations of nucleoside mix.


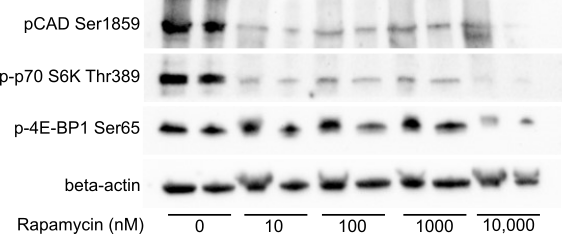


### Supplementary Figure S7. Related to Figure 5a.

T-cells were treated with the indicated concentrations of rapamycin for 48 hours and levels of mTORC1 phosphorylation targets were assayed by western blot.
